# A favorable outcome from dengue hemorrhagic fever: Teaching Hospital Kandy, Sri Lanka, May 2016

**DOI:** 10.3402/gha.v9.33186

**Published:** 2016-09-22

**Authors:** NDB Ehelepola

**Affiliations:** The Teaching (General) Hospital Kandy, Kandy, Sri Lanka

**Figure:**
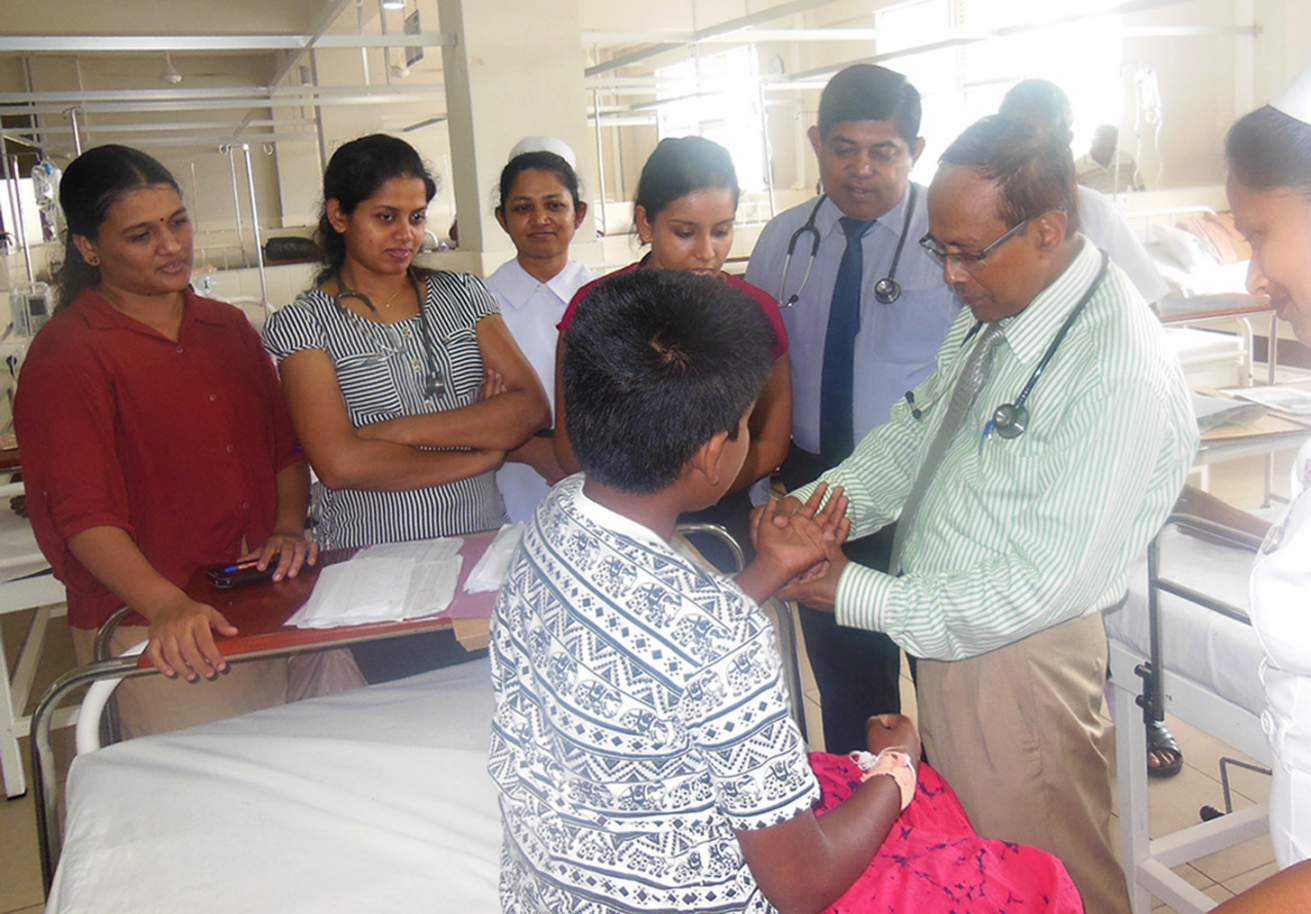


A consultant physician examines the rash on the forearm of a boy recovering from the critical phase of dengue hemorrhagic fever (DHF) at one of the internal medicine units of the Teaching Hospital Kandy, Sri Lanka, in late May 2016 during the usual ward rounds. Another doctor briefing him, has just said that this patient developed a prominent convalescent rash. The photographer also works at the same unit. Dengue is hyper endemic in Sri Lanka, and it is one of the major emerging public health problems locally as well as globally. Meteorological factors were proven to influence dengue transmission in Kandy (1) as well as Colombo (2), and there has been an increase in the number of dengue patients coming to us after the onset of first inter monsoon rains, with hot weather and narrowing diurnal temperature range. Although the incidence of dengue has risen, along with a rise in the proportion of DHF patients out of reported cases during the last decade, the case fatality rate has dropped compared to the past in Sri Lanka (3). This indicates an improvement of patient management during critical phases of dengue. This hospital and two other state-teaching hospitals cater for the great majority of severe dengue cases in the Kandy region and provide services free of charge. Free health care services and free education have immensely contributed to improving the health indices of Sri Lankans, although per capital total expenditure on health in Sri Lanka was very low – USD120 in 2013 (4). For example, life expectancy at birth in Sri Lanka in 2015 was 71.6 years for males and 78.3 years for females, better than most other developing nations in Asia and Africa (5). All hospital staff in this photograph are products of the Sri Lankan free education system.

*Ehelepola NDB* The Teaching (General) Hospital Kandy Kandy, Sri Lanka
